# Real-Time Detection of Human Growth Hormone Based on Nanoporous Anodic Alumina Interferometric Biosensor

**DOI:** 10.3390/s25041021

**Published:** 2025-02-09

**Authors:** Josep Maria Cantons, Laura K. Acosta, Pilar Formentin, Josep Ferré-Borrull, Akash Bachhuka, Lluis F. Marsal

**Affiliations:** 1 Department of Electronics, Electric and Automatic Engineering, Rovira i Virgili University (URV), 43007 Tarragona, Spain; josepmaria.cantons@urv.cat (J.M.C.); pilar.formentin@urv.cat (P.F.); josep.ferre@urv.cat (J.F.-B.); 2 Institute of Chemical Research of Catalonia (ICIQ), 43007 Tarragona, Spain; lkacosta@iciq.es (L.K.A.); abachhuka@iciq.es (A.B.)

**Keywords:** nanoporous anodic alumina, biosensing, human growth hormone, interferometry

## Abstract

Human growth hormone (hGH) is a polypeptide hormone that is synthesized and secreted by the anterior pituitary gland, whose excess is linked to acromegaly-causing pituitary adenomas while deficiencies are linked to disorders including short stature and Turner’s syndrome. This study investigates the real-time biosensing of hGH using a microfluidic optical biosensor based on reflectometric interferometry Fourier spectroscopy (RIFTS). The biosensing platform is based on a monolayer of nanoporous anodic alumina (NAA) fabricated following the two-step anodization method to produce pore sizes between 30 and 35 nm. The sensitivity of the nanostructure is improved by increasing the effective surface area by widening the pores to about 45 nm. NAA structures are then functionalized to make them selective to hGH. The sensing performance of the system shows a linear detection range from 12.5 µg/mL to 100 µg/mL with a detection limit of 10.6 µg/mL. This biosensing platform demonstrates the capability to detect high concentrations of human growth hormone using a cost-effective, fast, and portable biosensing system.

## 1. Introduction

Human growth hormone (hGH) is a polypeptide hormone synthesized and secreted by the anterior pituitary gland [1]. As a member of the GH gene family, which includes prolactin and the placental lactogens, hGH plays a pivotal role in growth and metabolic regulation [2]. Deficiencies of hGH during childhood can result in conditions such as short stature, chronic renal insufficiency, or Turner’s syndrome [3]. On the other hand, high concentrations of hGH, which are often caused by pituitary adenomas, can lead to pathological conditions such as gigantism or acromegaly (>2 µg/mL over a 24 h period) [4,5]. The development of efficient techniques for hGH detection has received a lot of attention in recent years. Several immunosensor-based technologies have been assessed; among these, studies by Ramanavicine et al. have shown that electrochemical, surface plasmon resonance, and electroacoustic chemiluminescence methods offer great sensitivity for the detection of hGH [6]. Other approaches include electrochemical measurements utilizing polymer to monitor hGH levels in biological fluid [7] and polymer membranes for regulated hGH release in therapeutic applications [8]. Additionally, hGH concentrations in flow-based assays have been detected by optical measurements utilizing porous silica structures [9].

Despite these advancements, there remains a demand for a fast, portable, and cheap biosensing platform that can integrate seamlessly with advanced technologies. Optical label-free biosensors have shown significant promise in meeting these requirements [10]. Among these, NAA-based biosensing platforms have shown immense potential. The optical properties, low-cost fabrication, and high effective surface area of NAA make this nanomaterial interesting for sensing and photonic applications [11]. The possibility of tuning the optical properties of NAA by changing the fabrication parameters, due to its highly effective surface area, makes it feasible for diverse sensing applications [12,13,14,15]. Previous studies have demonstrated real-time monitoring of biotinylated molecules in functionalized NAA [16] and real-time detection of various aqueous solutions using different NAA structures [17]. Further studies have used NAA-based measurement devices to monitor glycosaminidase [18] and the heat-shock protein 70, a critical parameter in cellular physiology [19]. In this study, we introduce a label-free biosensing platform based on functionalized nanoporous anodic alumina (NAA) for the detection of high concentrations of hGH. We leverage the high surface area and optical characteristics of NAA to detect variations in the effective optical thickness (EOT) in response to different hGH concentrations by taking advantage of Fabry–Pérot interferences caused by light exposure. Moreover, the integration of NAA into a flow cell makes feasible the real-time detection of different concentrations of hGH. We achieve real-time and selective hGH detection by functionalizing NAA to improve selectivity, meeting the demand for quick and effective biosensing solutions.

## 2. Materials and Methods

### 2.1. Materials

High-purity aluminum substrates (99.999%) with a thickness of 0.5 mm were purchased from Goodfellow Cambridge Ltd. (Huntingdon, UK). Ethanol (C_2_H_6_O), acetone (C_3_H_6_O), oxalic acid (C_2_H_2_O_4_), perchloric acid (HClO4), chromic acid (H2CrO4), hydrogen peroxide (H_2_O_2_), 3-Aminopropyltriethoxysilane (C_9_H_23_NO_3_Si), human serum albumin (HSA), and IgG were provided by Sigma Aldrich (St. Louis, MI, USA). Recombinant human growth hormone (hGH) and Recombinant Anti-human growth hormone antibody (anti-hGH) were supplied by Abcam. Glutaraldehyde was provided by ANAME. Double de-ionized (DI) water (18.6 MΩ, Milli-Q^®^) was used for all the solutions unless otherwise specified.

### 2.2. Fabrication of Nanoporous Anodic Alumina

**Figure 1:**
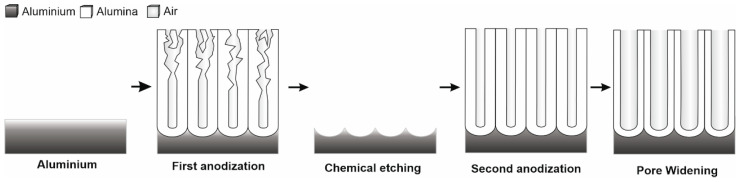
A graphical representation illustrating the two-step fabrication process of nanoporous anodic alumina nanostructures. The sequence begins with aluminum undergoing pore growth in a disordered pattern. This is followed by a chemical etching step, which creates nano-concavities that serve as initiation sites for further pore development. The process concludes with the formation of a highly ordered, self-organized nanopore structure.

Nanoporous anodic alumina (NAA) samples were fabricated through electrochemical anodization of aluminum (Al) using a two-step anodization method [20,21]. Before anodization, aluminum substrates were electropolished in a solution of ethanol–perchloric acid (HClO_4_) 4:1 (V:V) at 20 V for 8 min, with the current direction reversed each minute. The first anodization step was carried out in an aqueous solution of oxalic acid (H_2_C_2_O_4_) 0.3 M at 40 V and 5 °C for 20 h. Aluminum oxide layer was subsequently removed using a mixture of 0.4 M phosphoric acid (H_3_PO_4_) and 0.2 M chromic acid (H_2_CrO_4_) at 70 °C for 3 h, yielding a surface with a highly ordered pattern of nano-concavities. The resulting Al shows a surface with a highly ordered pattern formed by nano-concavities. The second anodization step was performed under the same conditions as the first one. The sample thickness was controlled by varying the anodization charge. In this case, eight samples (total anodized area = 4.02 cm^2^) were anodized simultaneously at 60 C to obtain a 5 µm pore length. A wet chemical etching in 5 wt % phosphoric acid (H_3_PO_4_) at 35 °C for 12 min resulted in a widening rate of about 1 nm/min for the pores. Figure 1 shows a complete schematic of the fabrication of the nanoporous anodic alumina substrates.

### 2.3. Scanning Electron Microscopy (SEM)

SEM images were performed with a Field Emission Scanning Electron Microscope (FESEM) (Scios 2, Thermo Fisher Scientific, Waltham, MA, USA) under a chamber pressure of 1 × 10^−4^ Pa, with electron beam voltages set between 30 kV and magnifications ranging from 50 KX to 250 KX, depending on the sample. The Everhart–Thornley detector (ETD) was used to detect secondary electrons (SEs).

### 2.4. Chemical Modification of NAA Surfaces

**Figure 2:**
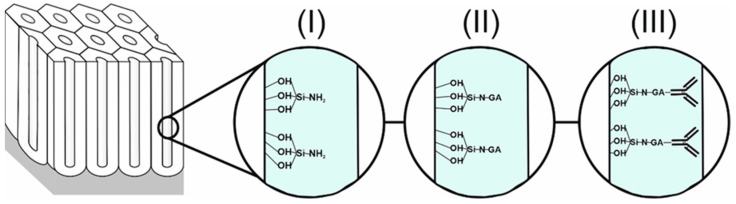
Graphical representation of the surface chemistry of nanoporous anodic alumina. The figure shows the different steps of the functionalization of the nanostructures as follows: (**I**) shows the APTES functionalization having a free NH_2_ group; this will then bind to the glutaraldehyde (**II**), and, finally, it will bind with the antibody of the human growth hormone (**III**).

The NAA surfaces were modified to create cross-linked amino groups [22]. These amino groups on the surface facilitate the immobilization of different biomolecules, making the biosensor selective and sensitive [23]. NAA samples were treated with 30% hydrogen peroxide at 115 °C for 30 min to create reactive hydroxyl groups on the surface. Post-treatment, samples were stored in the oven for 40–60 min at 60 °C to ensure the attachment of hydroxyl groups. The substrates were then washed with deionized water and dried in a nitrogen gas flow. Subsequently, the samples were exposed to a 1% (*v*/*v*) APTES solution in anhydrous toluene for two hours under N_2_ gas. After sequential washing with toluene, ethanol, and deionized water, the samples were dried under gas nitrogen flow and thermally cured at 110 °C overnight. The reaction with GTA involved a 10% (*v*/*v*) solution in anhydrous ethanol (Electron Microscopy Sciences) for 1 h at room temperature, ensuring cross-linking of aldehyde groups with amino groups. The samples were rinsed with ethanol and deionized water and dried with nitrogen. Finally, 10 µg/mL anti-hGH in phosphate-buffered saline (PBS) was incubated on the GA-modified structures overnight at 37 °C, forming amide bonds between GA’s aldehyde groups and the antibody’s amino groups [24]. Figure 2 details the functionalization process up to antibody immobilization.

### 2.5. Fourier Transform Infrared Spectroscopy (FTIR)

FTIR measurements were performed in the transmittance ATR mode using a Jasco FT/IR-6700 infrared spectrometer, covering the midinfrared region from 4000 to 450 cm^−1^, with a resolution of 4.0 cm^−1^ and 128 scans.

### 2.6. Reflectivity-Based Evaluation Throughout Fabry–Pérot Interferences

Reflectometric interference spectroscopy measures changes in the effective refractive index of NAA as molecules attach to the inner pore surface. For various NAA geometries, dimensions, and chemical compositions, NAA structures exhibit characteristic responses when they interact with light. These interferences arise from the optical path difference between the two beams reflected at the top and bottom interfaces of the NAA structure as in a Fabry–Pérot interferometer [25].

**Figure 3:**
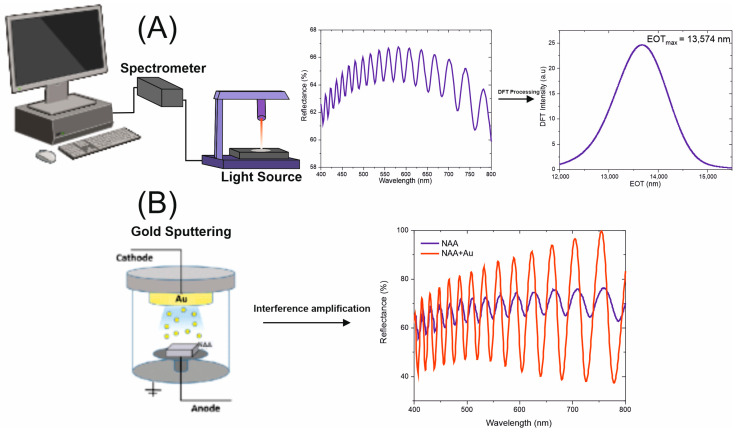
Interferometer setup for the interferometric measurements. (**A**) shows the characteristic Fabry–Pérot response from NAA and the data processing to obtain the effective optical thickness. (**B**) shows the effect of the gold sputtering of the NAA substrates.

The interferometric reflectance spectrum showed oscillations that were analyzed by a Fourier transform procedure that allowed us to calculate the effective optical thickness (EOT), whose equation is the following:

EOT_eff_ = 2 · n_eff_ · L (1)

where EOT_eff_ is the effective optical thickness, which is represented as a single peak after the Fourier transform processing, neff is the effective refractive index of NAA, and L is the thickness of the structure. The single peak that represents the EOT is useful for sensing as the molecules that bind to the NAA structure change the effective media [26]. Consequently, the effective refractive index causes a shift in the EOT_eff_ peak, see Figure 3A. NAA surfaces were sputter-coated with gold to improve the interferences, as shown in Figure 3B.

### 2.7. Optical Characterization of the NAA Substrates

Reflection spectra of NAA-GPCs were recorded from 400 to 800 nm at 2 nm resolution and 8° incidence angle using a PerkinElmer UV−visible−NIR Lambda 950 spectrophotometer. The real-time optical sensing was performed under continuous fluidic flow conditions at a fixed rate of 20 μL/min in real time using an acrylic plastic-based flow cell. Reflectance spectra from NAA-GPCs were acquired using a combined halogen light source (Spectligth DH-c) and optical-fiber spectrometer (Avantes, Apeldoorn, The Netherlands, AvaSpec-3648) Figure S3. Light from the source was focused onto the surface of NAA at normal incidence (0°) using a bifurcated fiber-optic probe with six illuminating waveguides assembled around one central reading waveguide. The optical probe was coupled to an optical lens to focus light within a ∼1 mm diameter circular spot on the top of NAA. The collection of reflected light was performed by the optical-fiber probe and guided to the spectrometer, which recorded one spectrum every 2 s with an integration time of 100 ms. Reflectance spectra were measured for the wavelength range of 500−900 nm. The resulting data were processed with a custom-made MATLAB R2020a program.

### 2.8. Surface Sputtering

Gold was sputtered on the samples at 0.05 mbar and 30 mA for 30 s, resulting in approximately 10 nm of gold overlayer on the NAA, using an AGAR Auto Sputter Coater Aname (Madrid, Spain).

## 3. Results and Discussion

### 3.1. Fabrication and Morphological Characterization of the Designed Nanostructures

**Figure 4:**
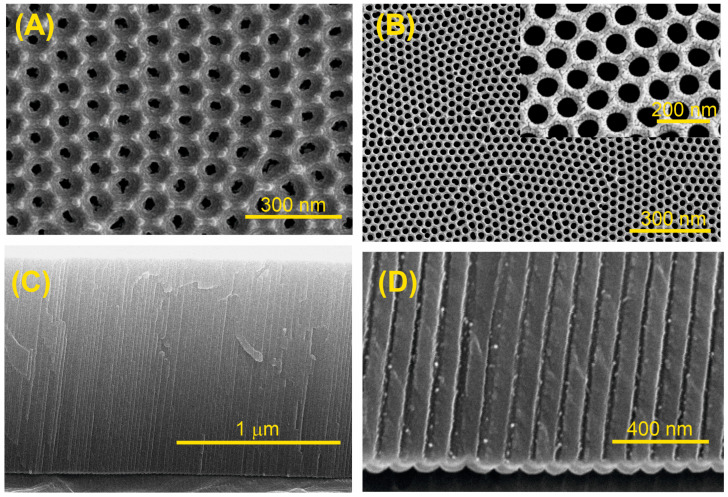
FESEM images of NAA structures: (**A**) Top view of self-ordered NAA with pore diameters of 33 ± 4 nm; (**B**) Top view of self-ordered NAA after a pore widening treatment, showing increased pore diameters of 44 ± 3 nm inset an amplified top-view zone showing the self-ordering of the nanopores; (**C**) Cross-section of NAA nanostructure showing a thickness of 5.2 ± 0.3 µm; (**D**) Cross-section image showing the cylindrical and parallel arrangement of the pores, along with barrier layer at the base.

Figure 4, Figure S1 and Figure S2 show representative FESEM images of the NAA structures, detailing the geometrical characteristics of the samples. The top-view image (Figure 4A) demonstrates a well-ordered hexagonal arrangement of pores, indicative of successful two-step fabrication. The fabricated samples exhibit a pore diameter of 33 ± 4 nm. Figure 4B shows the top view of the NAA structures after 12 min of pore widening, preserving the high degree of pore arrangement. The samples show an increased pore diameter of 44 ± 3 nm after chemical treatment. The NAA structure’s cross-sectional analysis, as displayed in Figure 4C, indicates a pore length of 5.2 ± 0.3 µm. Furthermore, Figure 4D highlights the cylindrical and parallel alignment of the pores, with a clearly defined barrier layer at the bottom of the structure.

### 3.2. Chemical Characterization of the Biofunctionalized NAA Substrates

**Figure 5:**
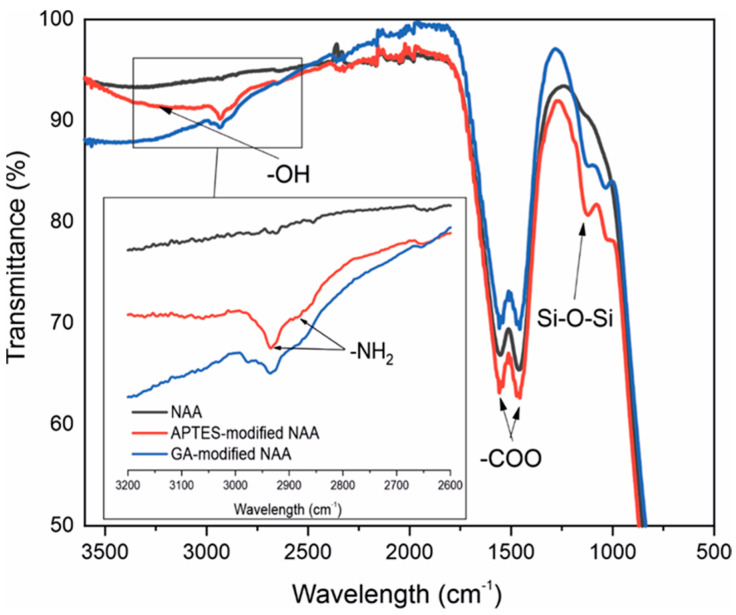
Transmission infrared spectra of NAA in different functionalization stages. Showing in black the spectra of NAA as fabricated. After the APTES functionalization, the NH_2_ and the Si-O-Si peaks appear in red. The inset shows the effect of the GA when it binds to the NH_2_.

The surface chemistry of the NAA substrate was characterized using FTIR to validate different stages of functionalization, such as APTES silanization and GA functionalization. The FTIR spectrum in Figure 3 and Figure S4 depicts distinct features at various wavenumbers, affirming the successful evolution of NAA during each functionalization step. The broad dip signal around 3300 cm^−1^ indicates hydroxyl group (-OH) stretching originating from alumina and hydrogen peroxide [27], indicating the presence of the Al–OH bond [28]. The intense double absorption peaks at 1459 cm^−1^ and 1555 cm^−1^ are attributed to the symmetric and asymmetric stretching of the carboxylate groups (-COO) of oxalic acid that coincides with the characteristic peaks of NAA [29]. The absorption at 1054 cm^−1^ signifies the formation of Si–O–Si links originating from APTES [30]. The inset in Figure 5 shows two additional peaks observed at 2854 cm^−1^ and 2925 cm^−1^ that correspond to the -NH_2_ groups [31]. Following GA treatment, the spectrum depicts two peaks indicative of CH_2_ deformation, with the peak at 2854 cm^−1^ disappearing, confirming the successful attachment of GA functional groups [32].

### 3.3. NAA’s Integration into the Flow Cell: Its Function and Assessment

The modified NAA structure was integrated into an acrylic flow cell to conduct sensing studies. A peristaltic pump was used to inject different hGH concentrations (from 12.5 to 100 µg/mL) at a constant flow rate of 20 µL/min. First, over the course of 20 min, a stable baseline was established using PBS. After that, a single concentration of hGH was injected into the flow cell for 40 min, during which time the maximum value of EOT was collected. Following the hGH injection, PBS flowed for 20 min to remove non-specifically bound molecules from the functionalized NAA structure.

### 3.4. Real-Time Optical Detection of Human Growth Hormone

To assess our biosensor’s response to hGH, concentrations of 12.5 µg/mL, 40 µg/mL, 50 µg/mL, 75 µg/mL, and 100 µg/mL in PBS buffer were used. The position of the FFT peak along the x-axis equals the refractive index and thickness of the NAA nanostructure. The reflectance spectra were recorded every second throughout the experiments. The data were then normalized using Equation (2), where EOT_o_ is the EOT signal obtained in the baseline and EOT_f_ is the signal obtained as a response, in this case, after 40 min.

(2) $$\frac{{\Delta EOT}_{f}}{{EOT}_{o}}=\frac{{EOT}_{f}-{EOT}_{o}}{{EOT}_{o}}$$

**Figure 6:**
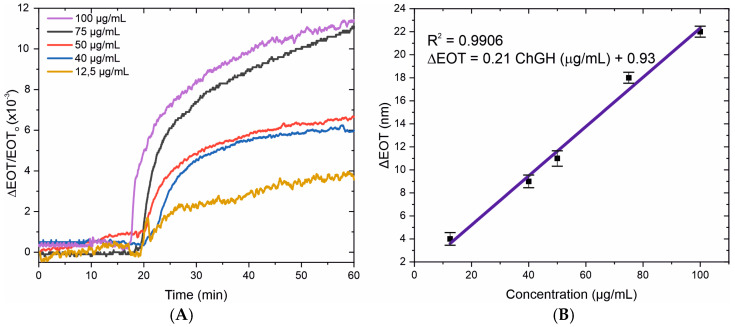
(**A**) Normalized EOT variation of the biosensor in the presence of different concentrations of hGH. (**B**) Linear regression of the biosensing response from 12.5 µg/mL to 100 µg/mL of hGH, showing a linear correlation of R^2^ = 0.991.

Figure 6A shows the normalized EOT change for each concentration, illustrating the real-time behavior and response of our biosensor to hGH. The binding interaction of the hGH with the anti-hGH antibody causes a measurable change in the EOT, as can be seen in either of the concentrations used (Figure 6A). The kinetics of antigen–antibody binding exhibit consistency with other biomolecule detection methods, including the Streptavidin detection study (0.5–50 µg/mL) [16]. Comparable binding-induced EOT changes were also shown in a different investigation that used porous silicon in a flow cell setup for hGH detection (0.0005–50 µg/mL) [9].

By calculating the difference between the maximum EOT value following the 40 min flow period and the baseline, the association between EOT shifts and hGH concentration was established. Figure 6B shows the linear behavior of the biosensor across the 12.5 µg/mL to 100 µg/mL range. The linear regression equation is expressed as ΔEOT (nm) = 0.21 ChGH (µg/mL) + 0.93, exhibiting a substantial correlation coefficient of R^2^ = 0.9906. The limit of detection (LOD) is established at 10.6 µg/mL, computed using the equation LOD = 3σ/s, where σ and s represent the standard deviation of the y-intercept and the linear regression slope, respectively. These findings highlight the biosensor’s sensitivity of 0.21 nm/[µg/mL] for the detection of hGH.

Notably, this study presents the possibility of detecting high concentrations of hGH that can be associated with acromegaly hormonal disorder. Our biosensor performance investigation demonstrates its comparability with existing biosensing platforms that are based on different detection techniques and designed to detect hGH. Our biosensor’s performance shows superior performance in several aspects compared to existing platforms that use diverse detection techniques for hGH measurements. These examples include the use of fluorophores that makes possible the fluorescent detection of hGH (0.1–1 µM), showing a linearity of R^2^ = 0.981, which means that that system is less linear than ours (R^2^ = 0.991) [33]. This linear behavior indicates that our biosensor provides more reliable and consistent detection across the tested concentration range. Others used electrochemical immunosensors, obtaining ranges from 0.1 to 100 ng/mL, which differs from our findings [34]. In this case, our sensor operates effectively within the higher concentration range relevant for the clinical diagnosis of the condition of acromegaly.

In another method, the authors used the absorbance of gold nanoparticles as a detection method for hGH, obtaining a sensitivity of 0.27 [a.u]/nM, which is comparable to our biosensor [35]. However, our biosensor’s optical approach offers a more straightforward and potentially less costly detection mechanism without the need for complex nanoparticle synthesis. Other authors have reported a method based on surface-enhanced Raman spectroscopy to detect the human growth hormone, where they found a precise method for the detection of this hormone [36]. They found an LOD of 0.082 nmol/L and a lower linearity of the sensor of R^2^ = 0.9831 compared to ours. There are other methods that use, for example, electrochemical impedance systems [7], where it seems that they use higher concentrations compared to ours (100 µg/cm^3^ to 100 mg/cm^3^), showing a linearity of 0.999 and an LOD of 2.97 × 10^−12^ g/cm^3^. A similar study to ours used the same method based on optical detection. The authors used porous silica as a sensing platform, getting a linear range from 0.0005 to 50 µg/mL and showing a linearity of R^2^ = 0.979 [9]. Our sensor, despite having a higher limit of detection, shows higher linearity, which indicates that our system provides more precise and accurate measurements over its operational range, leading to better performance quality and reliability in real-time applications.

Our biosensor’s performance demonstrates attributes of being remotely operative, cost-effective, user-friendly, and capable of detecting hGH in real time. Our findings demonstrate the feasibility of our biosensor for hGH detection, establishing a proof of concept. Future research will focus on expanding its applicability by evaluating its affinity for multiple biomolecules in parallel, thereby improving its robustness and practical utility. Long-term stability and multi-cycle testing across varying conditions are critical for practical implementation. Optimization of the biosensor could involve tuning the nanostructure’s geometrical properties to increase the effective surface area, thereby enhancing sensitivity. Adjusting pore size plays a crucial role in biosensor performance, as it influences sensitivity, linearity, responsivity, and selectivity. While larger pores improve biomolecule immobilization and analyte transport, excessively large sizes may reduce functional group density, affecting interaction efficiency. Conversely, smaller pores enhance selectivity but may slow down diffusion, impacting response time. Optimizing pore size, pore length, and the geometrical structure of NAA remains key to balancing these factors for improved biosensor performance.

### 3.5. Selectivity Test of the Biosensor

**Figure 7:**
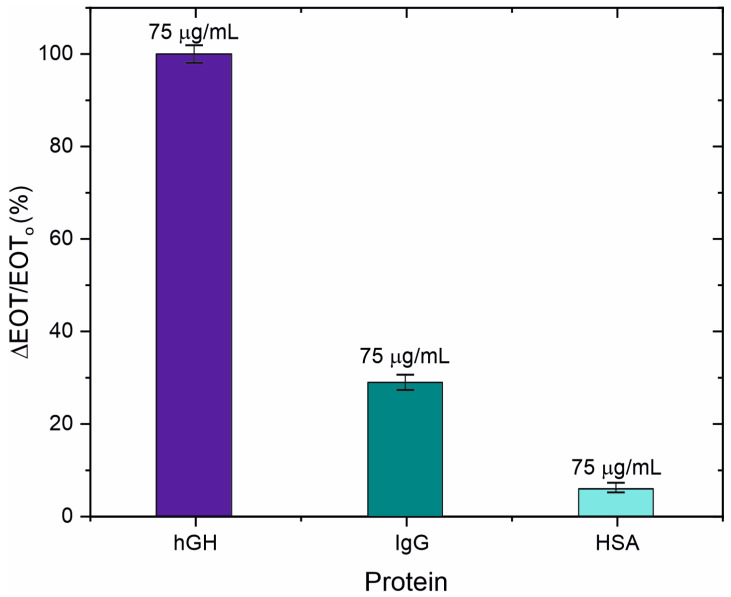
Selectivity test of the biosensor using two different proteins as a control: human serum albumin (HSA) and IgG.

To assess the selectivity of our biosensor, proteins human serum albumin (HSA) and immunoglobulin G (IgG) were tested at 75 µg/mL and compared with the target protein (hGH). After introducing each protein into the flow cell for 40 min, all unbound proteins were washed out with PBS for 20 min Figure S5. Figure 7 depicts the selectivity response of our biosensor. The biosensor’s capacity to discriminate between hGH and HSA was demonstrated by the low reaction to HSA, with a signal below 10%. Because IgG and the target biomolecule share certain structural similarities, the reaction to IgG was somewhat stronger. Despite this, the biosensor clearly showed that it could distinguish between hGH, IgG, and HSA, highlighting its selectivity for the target biomarker.

## 4. Conclusions

This study introduces, for the first time, a real-time label-free biosensor based on NAA to detect high concentrations of hGH. The functionalization of NAA nanostructures with APTES and glutaraldehyde facilitates the attachment of anti-hGH antibodies, making the biosensor highly selective to hGH. The biosensor demonstrated a linear detection range from 12.5 to 100 µg/mL with a limit of detection of 10.6 µg/mL. These findings can be further optimized by increasing the effective surface area of the used nanostructure to improve the sensitivity, LOD, and linear range of the biosensor. By doing this, the biosensor will be more sensitive, and then the values of detection will be closer to the early acromegaly values.

This work demonstrates NAA’s capacity to selectively recognize hGH even in the presence of other proteins, marking the first use of NAA as a real-time optical biosensor for hGH detection. Our findings demonstrate the biosensor’s negligible cross-reactivity and efficient separation of hGH from HSA and IgG. The competitive performance of our biosensor in comparison to other platforms, such as electrochemical immunosensors, fluorophore-based systems, and gold nanoparticle techniques, is highlighted by comparative analysis. Notably, our sensor demonstrates equivalent or superior sensitivity and improved linearity, proving its usefulness for high-precision diagnostics. Additionally, the technology holds great promise for adaption to a wide range of biomarkers, presenting broad potential applications in fields such as food technology and clinical analysis.

## Data Availability

The data that support the findings of this study are available from the corresponding author upon reasonable request.

## Supplementary Materials

The following supporting information can be downloaded at: https://www.mdpi.com/article/10.3390/s25041021/s1. Supplementary material about the increment of the pore diameter, including additional FESEM images, FTIR analysis of the NAA functionalized samples, and real-time monitoring of biomolecules for the selectivity test. (PDF). Figure S1: Top-view FESEM images of NAA samples; Figure S2: Top view and cross-sectional view of NAA samples; Figure S3: Real-time sensing setup for the detection of the selected biomolecules; Figure S4: FTIR spectra of NAA functionalized samples; Figure S5: Real-time monitoring of different biomolecules for the selectivity test.
