# Supplementary material for: Real-Time Detection of Human Growth Hormone Based on Nanoporous Anodic Alumina Interferometric Biosensor
Source: Sensors (Basel). 2025 Feb 9;25(4):1021. doi: 10.3390/s25041021 (PMC11859889; doi:10.3390/s25041021)
Supplement: Supplementary file 1 [file sensors-25-01021-s001.zip › sensors-3460247-supplementary.pdf]

Supporting Information

REAL-TIME DETECION OF HUMAN GROWHT HORMONE BASED ON NANOPOROUS ANODIC ALUMINA INTERFEROMETRIC BIOSENSOR

Josep Maria Cantons[1], Laura K. Acosta[2], Pilar Formentín[1], J. Ferré-Borrull[1], Akash Bachhuka[2] and Lluis F. Marsal[1]*

[1]Department of Electronic, Electric and Automatics Engineering, Rovira i Virgili University, Tarragona 43007, Spain

[2] Institute of Chemical Research of Catalonia (ICIQ), Tarragona, Spain 43007.

*E-mail: lluis.marsal@urv.cat

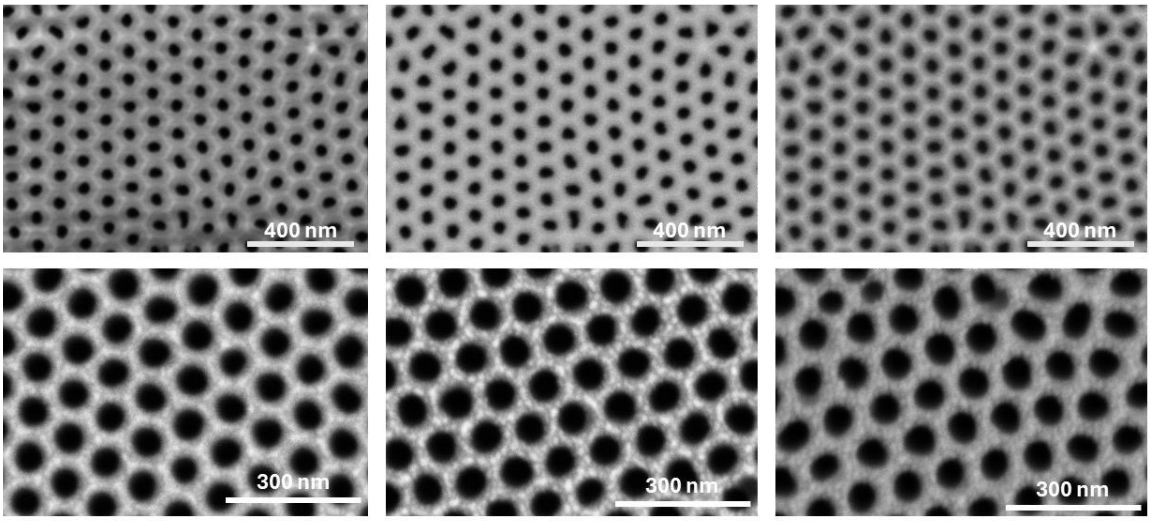

Figure S1: Top view FESEM images of NAA samples after second anodization. The three on top have a pore diameter of 32 nm average. The three on the bottom are after structural modification, pore widening, obtaining pores of around 42 nm in average.

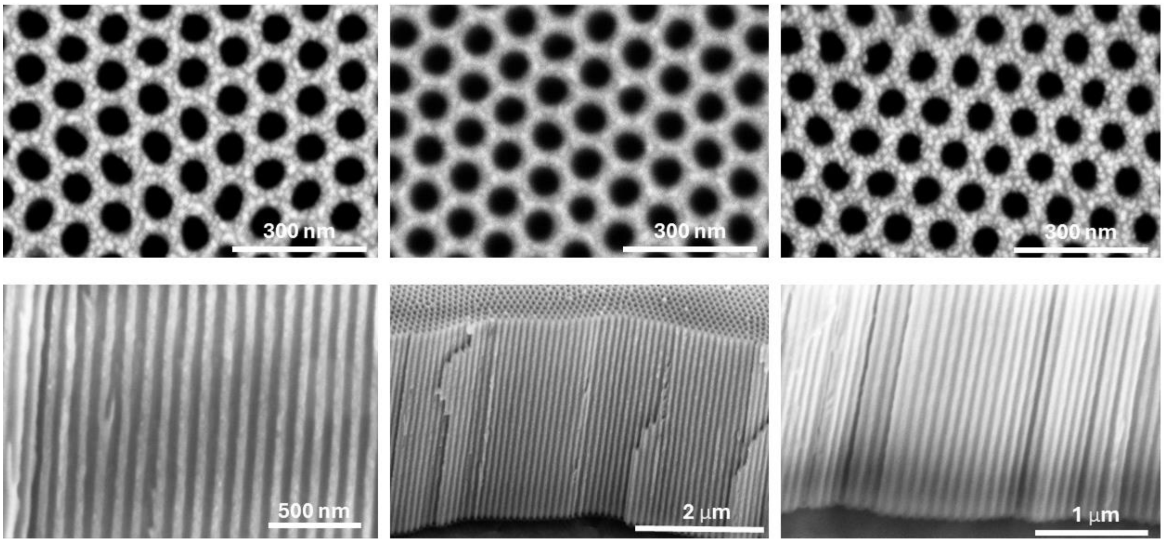

Figure S2: Top view FESEM images of NAA samples after pore widening and cross-sectional view of NAA. The three on top have a pore diameter of 43 nm average. The three on the bottom are after structural modification, pore widening, obtaining pores of around 42 nm in average.

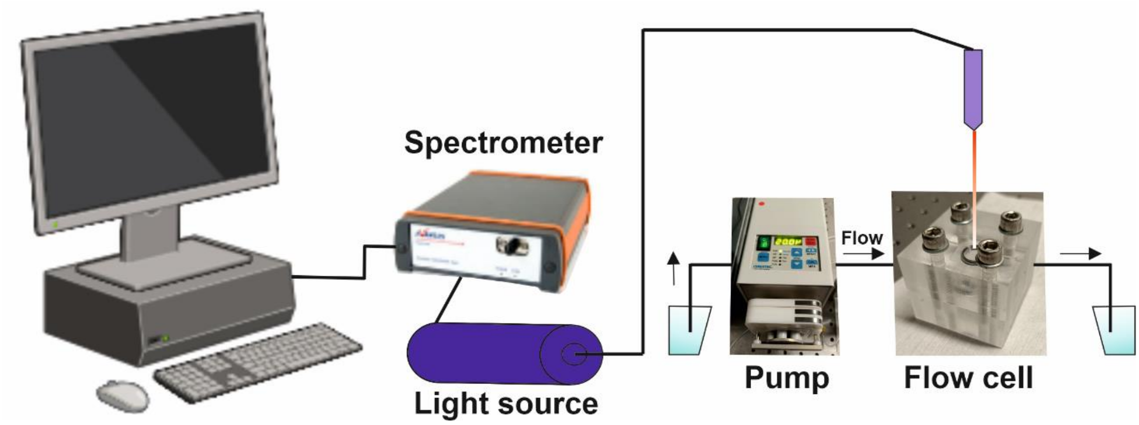

Figure S3: Set-up for the real-time sensing experiments for the detection of the targeted biomolecules.

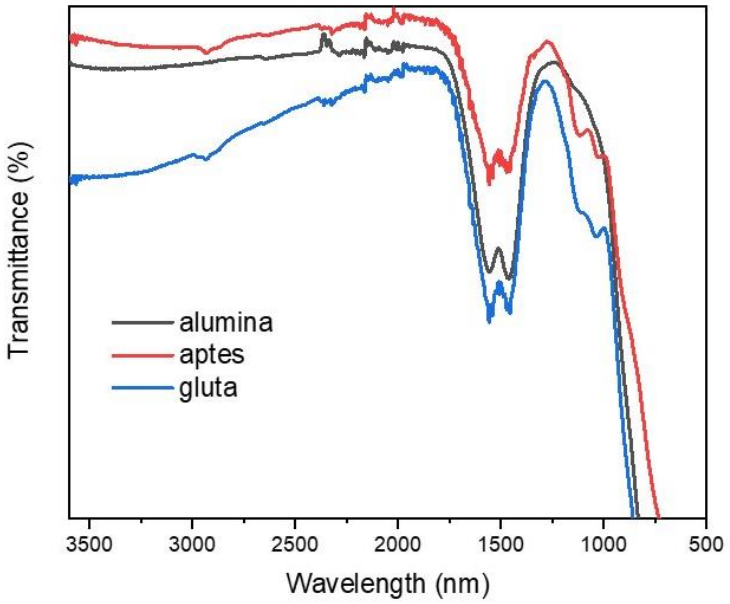

Figure S4: FTIR spectra of NAA samples at the first two stages of functionalization.

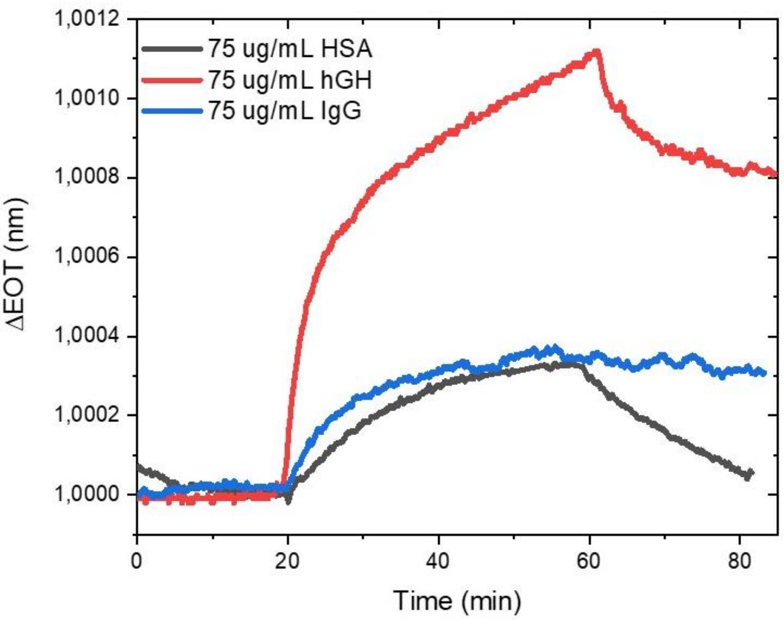

Figure S5: Real-time selectivity experiments using two different biomolecules and the human growth hormone.
